# Suggestions for political reparations for reproductive abuses against Black women

**DOI:** 10.3389/frph.2023.980828

**Published:** 2024-04-03

**Authors:** Micaela Stevenson

**Affiliations:** Department of Obstetrics and Gynecology, Medical College of Wisconsin, Milwaukee, WI, United States

**Keywords:** reproductive justice (RJ), reparations for historical injustices, reproductive health, access to contraception, access to fertility

## Introduction

Multiple scholars and activists have argued that Black women and other marginalized genders (i.e., transgender, gender nonconforming, and agender) are the most marginalized and minoritized people both in the United States and globally. The suffering Black women experience is broad from numerous microaggressions on a regular basis (1) as well as blatant macroaggressions (2) both of which cause compounding harm in the lives of Black women (2). These harms include but are not limited to higher rates of depression found in Black women experiencing higher rates of microaggressions as well as increasing levels of anxiety (2). These increasing levels of anxiety and depression are well known to impact cardiac function and have been implicated in cardiac disease.

Given the reproductive ability of the majority of Black women at some point in their life and the compounding of marginalization given the status as both women and as Black people, Black women are in a unique position to be victims of reproductive abuse. Discussions about reproductive abuses have been increasingly common as well and have included complicit and active actions of obstetrician gynecologists in the United States (3–5). Given the increasing discussion regarding reproductive abuses and understanding implicit bias in reproductive medicine, in this article, I hope to review these abuses to make the case for the need for reparations, followed by concrete suggestions for providing these reparations to Black women.

J. Marion Sims, the commonly cited father of obstetrics and gynecology, has been credited with designing the speculum that we currently use when treating patients as well as surgical techniques to repair fistulas developed secondary to childbirth (6, 7). While these discoveries remain important modernly, we cannot discount the fact that J. Marion Sims operated on enslaved women with neither their consent nor anesthesia, despite giving anesthesia to white women after having perfected this procedure. These women were found to serve as his surgical assistants after medical students and in training physicians no longer were able to stomach the thought of torturing these women or found that his experimentations were scientifically flawed and inappropriate to perform on humans (6). To add insult to injury, we credit J. Marion Sims with these discoveries despite the fact that they had been coopted from other physicians in the field and many women across the world had initiated the designs for these speculums prior (8).

While many of us can agree that operating on nonconsenting enslaved patients is against both good judgement and medical ethics, J. Marion Sims was applauded during his life and after for these accomplishments. Additionally, the field continued to engage in practices similar to these. Black women in the Southeast United States remained victimized by undergoing “Mississippi Appendectomies” in which they were sterilized against their will, without their knowledge, and when these were not medically indicated (9). Additionally, incarcerated women, whose very experience and existence in incarceration intentionally mimics that of American chattel slavery (10, 11) continued to experience forced sterilization until 2012 in the state of California (12). This, however, does not account for the number of women who have and continue to experienced coercion, undo influence, and incomplete information when undergoing sterilization procedures.

Contraception coercion is ever present. The fact that many physicians who deliver reproductive healthcare view contraception as a medical requirement causes us to discuss the benefits of contraception without discussing its risks. We also introduce our own bias into these discussions and The American College of Obstetrician Gynecologists openly recommends discussing the most effective contraceptive methods first, instead of first discussing the patient's goals for contraception (13), which leads to patient's being more likely to use long acting reversible contraception (LARC) (14) and takes control away from patients in being able to regain desired fertility in a time they desire without presenting to medical care (15). Patient's identities have been implicated in certain studies regarding the amount of education and time spent with patients, showing that patients are exposed to medical procedures for insertion of LARC—which carries risk—without proper consent (16). Because effectiveness over autonomy is valued, Black women report experiencing high rates of marginalization while seeking family planning services as well as undue pressure to utilize LARC methods—leading to dissatisfaction with these methods and early discontinuation (17) Obstetrician gynecologists have not only individually performed these acts of injustices, American societies have encouraged these injustices to take place. Calls to action in the past have been directed at supporting organizations in engaging in transformative practices and supporting justice for patient safety (18). Additional calls to action have been discussed including increased resident physician education (19), however educational actions alone are insufficient to remedy these issues. Given the tangible harm that has occurred both by individuals and through reproductive societies at large with their complicit behavior, specific political actions and reparations must be considered to remedy this harm.

These can be difficult to define, promote, and enact. However, in order to generate the most good, professional societies can specifically target political actions in which they can engage to promote equity and attempt to redefine a new relationship that this field may hope to have with Black women who seek our services. The political weight that many professional reproductive health colleges and societies hold are well known. Many obstetrician gynecologists participate in political lobbying given the highly political nature of the work we engage in. Many women's health advocacy and lobbying projects have been led by obstetrician gynecologists including maternal and newborn health initiatives (20) and obstetrician gynecologists societies have argued for an integral role in advocacy for patients’ reproductive health (21).

Specific political reparative actions can revolve around using a reproductive justice approach. This specific perspective revolves around understanding that each person has the right to have or not have children and the right to parent children in safe environments (18). This approach has been specifically developed by Black women, including scholar activist Loretta Ross, and reinforced with ongoing literature and works by scholars such as Kimberle Crenshaw and Angela Davis. This perspective is designed to understand the specific struggles of Black women and to address their concerns from a perspective that will help the most.

## Specific steps for action

### Lobbying to change medicaid sterilization policies

The American College of Obstetricians and Gynecologists has published its stance on revising Medicaid policies to ensure that people can access sterilization without the need for waivers to increase access to sterilization. However, specifically introducing policies and engaging in ongoing lobbying to ensure that this action is passed. Lobbying coalitions have been proven to be an effective strategy for leading to change (22). While it may appear paradoxical to increase access to sterilization, the pendulum has swung too far in the opposite direction and does not allow there to be improved access to one of the most effective forms of contraception through sterilization. Ongoing agitation is required to allow there to be access to this method. Because obstetrician gynecologists have caused the requirement for this insurance mandate through inappropriate and forced sterilization, it is the responsibility of governing bodies and other professional societies to ensure that we continue using our political power to increase ongoing access when it has caused the absence of this access. These same lobbying tactics should be taken up by additional state and national level reproductive medicine societies. Studies have argued that by removing Medicaid sterilization policies will significantly increase access to sterilization by over 30%, would decrease unintended pregnancies by thousands, and would save millions of dollars (23).

### Call for and continue ongoing lobbying for requirements for fertility treatment in all health insurance policies in the United States

The reproductive abuses that Obgyns have caused to Black women has often led to infertility. Therefore, one way to repay these groups is to attempt to offer medical treatment that will assist them in conceiving again. Given that Black women suffer disproportionately higher rates of infertility and have disproportionately lower access to fertility treatments secondary to cost (24, 25), it is integral that to reduce these barriers, we call for coverage for infertility. Many professional reproductive health societies have yet to call and lobby for insurance plans to cover infertility treatment in all medical insurance plans. Therefore, these societies should use their political pull and power for ongoing lobbying of infertility coverage. Insurance mandates have been shown to increase access for marginalized people to effective, infertility treatment and we can therefore believe that expanding this across all insurance plans will continue to improve access fertility treatment (25). As many people in the United States, including Black people, use Medicaid as their primary health insurer, ensuring infertility coverage is included in Medicaid plans is vital to ensuring increased access to care for all patients, especially the most marginalized patients.

### Call for defunding of police and carceral systems

Carceral systems and modern policing are rooted in racist, chattel slavery in the United States (26, 27). These also dis To truly be able to move effectively provide reparations, we must recognize that the prison systems in which so many people have been forcibly sterilized are rooted in racism and other forms of oppression. Therefore, aligning with groups that have started organizing to abolish and defund policing in the United States is integral. While this alone is unlikely to be the sole solution, given that sterilization abuses occur outside of these settings. However, it would eliminate a hierarchy in which incarcerated people are at the mercy of systems designed to harm them and prevent those systems from sterilizing them against their will and promoting sexual abuse. While the American College of Obstetricians and Gynecologists has expressed alignment with ending sterilization of incarcerated women due to this hierarchy (28), ongoing lobbying will assist in eliminating the additional abuses that occur in these settings.

### Begin the process of defining the financial reparations that may be required

In discussing reparations, one commonly used method of righting wrongs includes providing monetary compensation for the damage which has been done. However, it is difficult to specifically define the cost of intentional iatrogenic loss of fertility as well as the cost of psychological harm and trauma physicians may be responsible for under the watchful eye of a governing body. Without asking Black communities and individual Black people who have lost their fertility due to our wrongs, we cannot know what the monetary value of this loss may be. Therefore, research should be conducted to assess what financial reparations might feel most appropriate to individuals and their communities. That research should then be used to inform specific next steps which can be taken to compensate these groups. Some examples of how to appropriately assess how to provide these reparations can be found in assessing what reparations were offered after the Tuskegee Syphilis Experiment (29, 30). Many additional steps have been discussed about financial reparations for the Guatemalan Sexually Transmitted Infection Experiments 31), but these have yet to be enacted. Attempting review the proposed model and try to enact these is promising and may help to create positive relationships. Importantly, multiple groups of people receive reparations from various countries or organizations globally, including people Indigenous to the United States and Jewish people who had family members victimized in the Holocaust. These have been found to be acceptable by the United Nations and therefore, offering similar reparations on the same basis to Black women will likely be both acceptable and welcome—given widespread belief that Black people deserve reparations in Black communities and decades of discussion arguing for the need for these.

## Conclusion

Reparations are a requirement to move forward and commonly promote healing. Given the long history of abuses that have occurred against Black women and other people who can become pregnant, obstetrician gynecologists should consider the role that individually we have played and overall, the role that the field has played in marginalizing this population. We have a responsibility to try to do better for our patients and for the sake of our colleagues and for ourselves. These reparations may not be the final solution. However, given the known benefits of transformative justice, we must try something to move forward to reduce the pain and suffering our patients experience at our hands.
